# Effectiveness and safety of lubiprostone after switching from stimulant laxatives in elderly patients with chronic constipation

**DOI:** 10.1002/jgh3.12956

**Published:** 2023-08-11

**Authors:** Masaki Maruyama, Suguru Miida, Toshihiro Sato, Takazumi Kimura, Azuma Watanabe, Hideo Watanabe, Masafumi Nishizawa, Kyohei Horikawa, Takahiro Kajiwara, Yusuke Karasawa, Yuko Hasebe, Kazutaka Nozawa, Shuji Terai

**Affiliations:** ^1^ Department of Internal Medicine Kashiwazaki General Hospital and Medical Center Kashiwazaki Niigata Japan; ^2^ Sato Clinic Kashiwazaki Niigata Japan; ^3^ Kimura Internal Medicine Clinic Yokohama Kanagawa Japan; ^4^ Department of Gastroenterology Kameda Daiichi Hospital Niigata Japan; ^5^ Department of Surgery Watanabe Hospital Matsuyama Ehime Japan; ^6^ Department of Internal Medicine Minamisanriku Hospital Motoyoshi‐gun Miyagi Japan; ^7^ Wakamatsuen Health Care Facility for the Elderly Okinawa Japan; ^8^ Lotus Care Center, Health Care Facility for the Elderly Chiba Japan; ^9^ Medical Affairs, Viatris Pharmaceuticals Japan Inc. Tokyo Japan; ^10^ Division of Gastroenterology & Hepatology, Graduate School of Medical and Dental Sciences Niigata University Niigata Japan

**Keywords:** constipation, drug therapy, elderly, laxatives, lubiprostone

## Abstract

**Background and Aim:**

Stimulant laxatives may cause electrolyte abnormalities, dehydration, and abdominal pain; their long‐term use can lead to tolerance and subsequent refractory constipation. We investigated the effectiveness, safety, and quality of life after switching from stimulant laxatives to lubiprostone in elderly patients with chronic constipation (CC).

**Methods:**

This multicenter, interventional, open‐label, single‐arm, before‐and‐after comparison study enrolled 99 Japanese patients aged 65–90 years with CC who took stimulant laxatives for ≥2 weeks prior to switching to lubiprostone monotherapy.

**Results:**

The mean ± SD spontaneous defecations at Week 1 of 7.8 ± 6.2 times/week was not significantly different from that at baseline (8.3 ± 4.7). Spontaneous defecations were significantly reduced at Weeks 2 (−1.5 ± 4.0, *P* < 0.001) and 4 (−1.5 ± 3.7, *P* < 0.001). The Bristol Stool Form Scale score did not change from baseline (4.7 ± 0.9) at Weeks 1 (4.5 ± 1.3) or 4 (4.3 ± 1.3), but it did at Week 2 (4.3 ± 1.5, *P* < 0.05). The Patient Assessment of Constipation Quality of Life questionnaire score increased (0.36 ± 0.07, *P* < 0.001) after 28 days. Nausea was the only symptom that worsened from baseline and was the most frequently reported adverse drug reaction (15.2%).

**Conclusion:**

Switching to lubiprostone monotherapy for CC was not associated with significant concerns in short‐term spontaneous defecation frequency and safety, but it might affect the efficacy and patient quality of life over 2 weeks. Careful treatment strategies facilitating gradual switching to lubiprostone monotherapy may be needed in patients using stimulant laxatives.

## Graphical abstract

This study is a multicenter, interventional, open‐label, single‐arm, before‐and‐after comparison study enrolled 99 Japanese patients aged 65–90 years with chronic constipation who took stimulant laxatives for ≥2 weeks prior to switching to lubiprostone monotherapy. Lubiprostone monotherapy was substantially effective and safe when used as switching therapy for elderly patients who were constipated and had been treated with stimulant laxatives over 2 weeks.

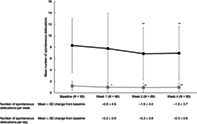

## Introduction

Chronic constipation (CC) is defined as a state in which feces that should be eliminated from the body cannot be passed in sufficient quantity and comfort.^1^ Current estimates indicate that the prevalence of constipation‐related symptoms in Japan is 4.4% in women and 2.5% in men.^2^ Japanese studies have identified age and frailty as risk factors for constipation.^3^, ^4^ Given Japan's rapidly aging society, the population of Japanese elderly patients with constipation is growing.^2^ The proportion of female patients with constipation is higher than male patients among those aged 60–69 years. However, the prevalence increases with age in both sexes, and the sex difference disappears among those older than 70 years of age.^2^ Elderly patients with constipation report poorer mental status, higher psychological distress, lower physical functioning, poorer general health perception, and higher bodily pain scores than non‐constipated elderly patients.^5^, ^6^ Furthermore, underdiagnosed and undertreated constipation leads to poor quality of life and increased healthcare spending,^7^ and even patients who receive treatment report not being satisfied with the outcomes.^8^ Thus, the recognition and appropriate management of CC are essential for maintaining the quality of life in elderly constipated patients.^9^

The current standard treatment for CC consists of dietary and lifestyle guidance (i.e., increased fluid, fiber, and exercise) followed by pharmacologic treatment when improvement with lifestyle changes is challenging.^10^ The most frequently used drugs in Japan are osmotic laxatives, such as magnesium oxide, and stimulant laxatives, such as senna (sennoside). The evidence‐based clinical practice guidelines for chronic constipation released in 2017 recommend primarily using osmotic laxatives and intestinal secretagogues, such as lubiprostone and linaclotide, with stimulant laxatives being next in line and administered as needed or on a short‐term basis.^1^ The American Gastroenterological Association's guidelines also recommend lifestyle guidance and administration of osmotic laxatives as first‐line constipation treatment and recommend using stimulant laxatives only when necessary.^11^ However, there is concern that osmotic laxatives such as magnesium oxide, which is the most commonly used, may cause hypermagnesemia in patients taking them for a long term or in elderly patients with impaired renal function. Stimulant laxatives are known to cause side effects, including electrolyte abnormalities, dehydration, and abdominal pain, and long‐term use can lead to tolerance, habituation, and subsequent refractory constipation.^12^, ^13^, ^14^ Anthraquinones are widely used in Japan, but no randomized controlled trials have been conducted to evaluate their efficacy and safety in patients with CC. Furthermore, the long‐term use of anthraquinone derivatives is reportedly linked to the development of melanosis coli, which may be a risk factor for colorectal cancer.^15^, ^16^ When considering reducing serious treatment side effects, special attention should be paid to elderly patients with CC. Lubiprostone, a bicyclic fatty acid compound that activates the type‐2 chloride ion channel present in the mucosal epithelial cells of the small intestine, promotes the secretion of electrolytes and fluid into the intestinal tract, ultimately leading to defecation.^17^ A Phase III clinical trial (trail registration: Japan Registry for Clinical Trials; identifier number jRCT2080220730) conducted in Japan for patients with CC showed that after 1 week of treatment, lubiprostone significantly increased the weekly average number of spontaneous defecations (change from baseline, +3.7 times/week).^18^ Long‐term data from the Phase III clinical study and a prospective open‐label study confirmed that lubiprostone was consistently effective over 48 weeks of administration.^18^, ^19^ Additionally, the Phase III study reported a significant increase in quality‐of‐life questionnaire scores with long‐term treatment.^18^

Considering the issues associated with the long‐term use of stimulant laxatives, ideally, regular administration of these drugs should be avoided by switching to other safer medications such as lubiprostone, especially in elderly patients. However, to date, few studies have investigated the effectiveness and safety of switching treatment from stimulant laxatives. The current study aimed to investigate the effectiveness and safety of switching from stimulant laxatives to lubiprostone monotherapy in elderly patients with CC.

## Materials and methods

### Study design

This was a multicenter, interventional, open‐label, single‐arm, before‐and‐after comparison study conducted in Japan at eight sites from 22 October 2020 to 22 September 2021. Before conducting the study, the study protocol was reviewed and approved by the third‐party Certified Review Board (CRB) of the Hattori Clinic (CRB3180027). The CRB of the Hattori Clinic is a research review committee that is approved by the Japanese Ministry of Health, Labour and Welfare, in accordance with the Clinical Research Act. The role of the CRB is to review and approve clinical research conducted under this Act. Written consent was obtained from all patients before enrollment in the study, and the study complied with the Clinical Research Act and related regulations. This study was funded by Mylan EPD Ltd., which merged during the study period to become Viatris Pharmaceuticals Japan Inc. Employees of Viatris Pharmaceuticals Japan Inc. were responsible for the independent analysis and interpretation of the data in this study.

Two study drug doses were used: lubiprostone capsules of 12 and 24 μg. The study treatment consisted of 24 μg of lubiprostone taken twice daily, after breakfast and dinner, for 28 days. If any adverse event was observed during the daily dose administration of 48 μg, the dose could be reduced to one capsule of 24 μg once daily, either after breakfast or dinner, or one capsule of 12 μg after breakfast and one after dinner, starting the day after the onset of the adverse event.

The diagnostic criteria for CC stated in the Evidence‐Based Clinical Practice Guidelines for Chronic Constipation 2017 were used to establish the diagnosis of constipation in this study.^1^ Constipation was diagnosed based on the Rome IV criteria,^20^ that is, if two or more of the following six criteria were met: (i) straining during more than one‐fourth (25%) of defecations; (ii) lumpy or hard stools (Bristol Stool Form Scale score [BSFS] 1–2) during more than one‐fourth (25%) of defecations; (iii) sensation of incomplete evacuation after more than one‐fourth of defecations; (iv) sensation of anorectal obstruction/blockage during more than one‐fourth (25%) of defecations; (v) manual maneuvers to facilitate more than one‐fourth (25%) of defecations (e.g., digital evacuation, support of the pelvic floor); and (vi) fewer than three spontaneous bowel movements per week. For the diagnosis of CC, symptoms must have been present for at least 6 months, and the above criteria must have been met for the last 3 months.

### Patients

The patients included were inpatients and outpatients with CC between the ages of 65 and 90 years who were treated with stimulant laxatives for at least 2 weeks prior to switching to lubiprostone monotherapy and could discontinue stimulant laxatives during the study period. Patients were excluded if they had severe cardiopulmonary complications, had confirmed or suspected bowel obstruction, had a history of hypersensitivity to lubiprostone, participated in other clinical trials and received the study medication within 1 month of starting lubiprostone, had been administered lubiprostone within 3 months of study participation, or had moderate or severe hepatic dysfunction or severe renal dysfunction.

### Study endpoints

The primary endpoint was the number of spontaneous defecations per week (times/week) at Week 1 after lubiprostone administration. The secondary endpoints were as follows: the number of spontaneous defecations (times/week, times/day) at Weeks 2 and 4; time to first spontaneous defecation after lubiprostone administration; change in Patient Assessment of Constipation Quality of Life (PAC‐QOL) questionnaire score^2^ at day 28 of lubiprostone administration; change in BSFS scores^1^, ^11^; and change in original scores for subjective symptoms such as abdominal pain, bloating, nausea, vomiting, depressed mood, anorexia, straining during defecation, and sensation of incomplete bowel movement at day 28 (each symptom was rated from 1 to 5).

### Safety

The safety endpoints included the frequency of adverse events and adverse drug reactions that occurred after the start of the lubiprostone administration.

### Data collection

After obtaining written consent, the following information was obtained: patient background, PAC‐QOL, defecation status, and concomitant use of the study treatment during the screening period (−9 days to −1 day before the start of the study treatment). Data for defecation status, concomitant medications/adjunctive therapies, study treatment, patient diary for defecation (including BSFS), and adverse events were collected every 7 days after starting study treatment. PAC‐QOL was collected only 28 days after the study treatment started.

### Statistical analysis

The proportion of patients with ≥3 bowel movements during the first 7 days of treatment with the study drug was assumed to be 60%. When the number of patients was set at 143, the 95% confidence interval for the percentage of patients with ≥3 bowel movements was ±8%, based on the approximation method using *z* statistics. Thus, the target sample size was set at 150 patients with CC with an expected dropout rate of 5%.

The full analysis set was defined as all eligible patients who received study treatment, excluding those who did not comply with the Clinical Research Act and those with no collected data. The per‐protocol set was defined as the full analysis set excluding patients who deviated from the research protocol. The safety analysis set was defined as patients who received at least one dose of the study treatment.

Summary statistics were calculated for patient background information, including mean ± SD for continuous data and *n* (%) for categorical data. Summary statistics were also calculated for the number of spontaneous defecations at each evaluation time point after lubiprostone administration and the change from pre‐administration. Paired *t*‐test and Wilcoxon's signed‐rank test were performed. Clopper–Pearson's exact confidence intervals were also calculated.

Summary statistics were calculated for the number of bowel movements before taking lubiprostone, PAC‐QOL, BSFS scores, defecation symptom scores, and change from pre‐treatment at each evaluation time point after lubiprostone administration. Paired *t*‐test and Wilcoxon's signed‐rank test were performed. The number and percentage of adverse events were calculated and tabulated. All statistical analyses were performed using SAS version 9.4 or higher (SAS Institute Japan Inc., Tokyo, Japan).

## Results

### Patients

**Figure 1:**
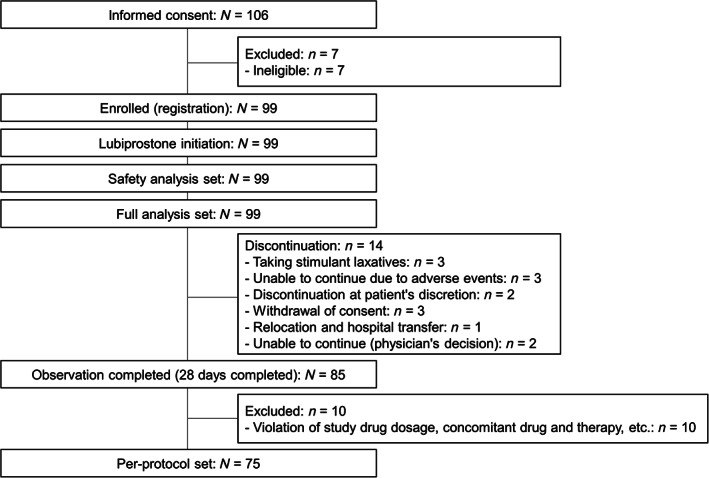
Patient flowchart.

Of the 106 patients who provided informed consent, 99 were enrolled and initiated lubiprostone treatment. Fourteen patients discontinued the study. The most common reasons for study discontinuation were taking stimulant laxatives, adverse events, and withdrawal of consent in three patients each. Eighty‐five patients completed the 28‐day observation period (Fig. 1). The characteristics of the enrolled patients are shown in Table 1. The most prescribed laxative as pretreatment was senna glycoside (71.7%), followed by sodium picosulfate (28.3%).

**Table 1:** Baseline patient demographic and clinical characteristics (full analysis set)

Variables	
Total, *n* (%)	99 (100.0)
Sex (female), *n* (%)	62 (62.6)
Age (years), mean ± SD	80.4 ± 5.8
Body mass index (kg/m^2^)^†^, mean ± SD	23.5 ± 4.2
Duration of constipation (months)^‡^, mean ± SD	129.1 ± 110.7
Pretreatment^§^, *n* (%)	99 (100.0)
Senna glycoside	71 (71.7%)
Sodium picosulfate	28 (28.3%)
Other	2 (2.0%)
Comorbidity, *n* (%)	77 (77.8)
Endocrine metabolic disease	19 (19.2)
Neurological disorder	13 (13.1)
Collagen disease	1 (1.0)
Neurodegenerative disease	1 (1.0)
Psychiatric disorder	12 (12.1)
Other	61 (61.6)
PAC‐QOL^¶^, mean ± SD	0.60 ± 0.05

^†^ *n* = 97.

^‡^ *n* = 79.

^§^ More than one laxative allowed.

^¶^ *n* = 93.

PAC‐QOL = Patient Assessment of Constipation Quality of Life.

### Primary endpoint

The mean ± SD number of spontaneous defecations after 1 week of treatment with lubiprostone was 7.8 ± 6.2 times/week, which was not significantly different from baseline (8.3 ± 4.7 times/week) (mean change, −0.6 ± 4.9, *P* = 0.261).

### Secondary endpoints

**Figure 2:**
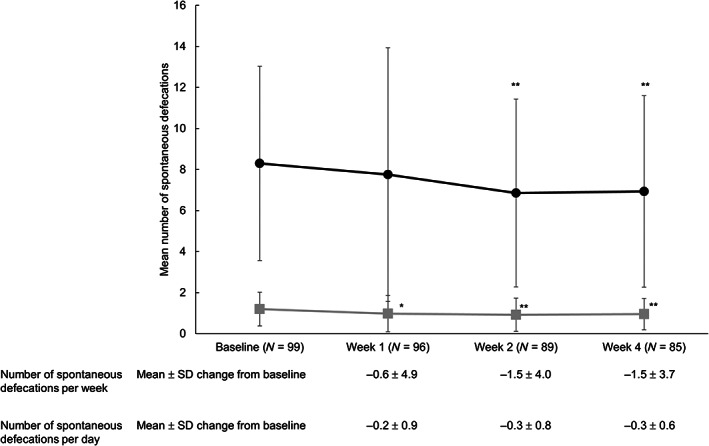
Transition in mean number of spontaneous defecations and mean change from baseline of spontaneous defecations. *P < 0.05, **P < 0.001 (paired *t*‐test). 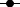, number of spontaneous defecations per week; 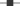, number of spontaneous defecations per day.

Significant decreases in the number of spontaneous defecations per week from baseline at Weeks 2 and 4 were −1.5 ± 4.0 and −1.5 ± 3.7, respectively (mean ± SD change from baseline, both *P* < 0.001) (Fig. 2). The average spontaneous defecations per day at Week 2 and 4 were 0.9 ± 0.8 and 1.0 ± 0.8, respectively, with a significant reduction from baseline (Week 2, −0.3 ± 0.8; Week 4, −0.3 ± 0.6; both *P* < 0.001) (Fig. 2). The mean ± SD time from the first lubiprostone dose to the first spontaneous defecation was 24.9 ± 41.4 h (data not shown).

**Figure 3:**
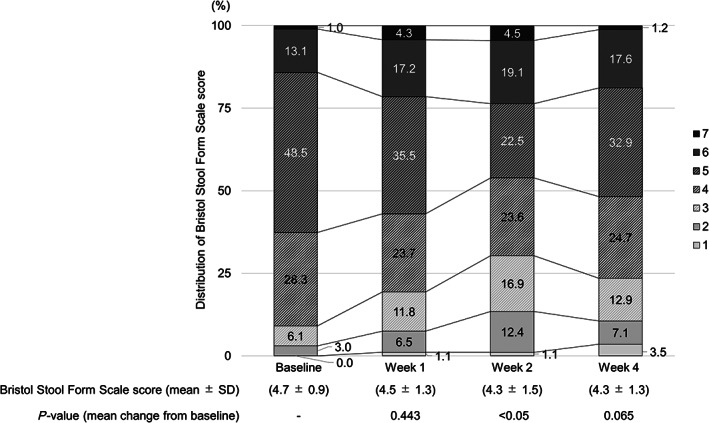
Transition in the distribution of Bristol Stool Form Scale scores. Wilcoxon's signed rank‐test.

The PAC‐QOL questionnaire score (mean ± SD) significantly changed from 0.60 ± 0.05 at baseline to 0.95 ± 0.07 in 28 days (mean change, 0.36 ± 0.07, *P* < 0.001) (Table 2). The BSFS score at baseline was 4.7 ± 0.9, and it did not change significantly at Week 1 (4.5 ± 1.3) or 4 (4.3 ± 1.3) relative to baseline, but a significant change was noted at Week 2 (4.3 ± 1.5, *P* < 0.05, Fig. 3). The symptom score was significant only for nausea (Table 3).

**Table 2:** Patient Assessment of Constipation Quality of Life total and dimension scores

	Baseline score (mean ± SD)	Day 28 (mean ± SD)	Change from baseline (mean ± SD)	*P*‐value*
No. of patients	93	90	85	
Total score	0.60 ± 0.05	0.95 ± 0.07	0.36 ± 0.07	<0.001
Physical discomfort	0.35 ± 0.05	0.67 ± 0.09	0.30 ± 0.09	<0.001
Psychosocial discomfort	0.26 ± 0.04	0.32 ± 0.05	0.05 ± 0.05	0.371
Worries/concerns	0.46 ± 0.06	0.80 ± 0.09	0.35 ± 0.08	<0.001
Satisfaction	1.66 ± 0.11	2.52 ± 0.10	0.91 ± 0.14	<0.001

* Paired *t*‐test.

**Table 3:** Median change in symptom scores from baseline to 28 days after lubiprostone initiation

	Baseline median (interquartile rage)	Day 28 median (interquartile rage)	Change from baseline median (interquartile rage)	*P*‐value*
Abdominal pain	5.0 (4.0, 5.0)	5.0 (4.0, 5.0)	0.0 (0.0, 0.0)	0.720
Abdominal distension	5.0 (4.0, 5.0)	5.0 (4.0, 5.0)	0.0 (0.0, 0.0)	0.482
Nausea	5.0 (5.0, 5.0)	5.0 (4.0, 5.0)	0.0 (−1.0, 0.0)	<0.001
Vomiting	5.0 (5.0, 5.0)	5.0 (5.0, 5.0)	0.0 (0.0, 0.0)	0.080
Depression	5.0 (4.0, 5.0)	5.0 (5.0, 5.0)	0.0 (0.0, 0.0)	0.173
Anorexia	5.0 (4.8, 5.0)	5.0 (4.0, 5.0)	0.0 (0.0, 0.0)	0.446
Straining	4.0 (3.0, 5.0)	5.0 (3.0, 5.0)	0.0 (−0.8, 1.0)	0.669
Sensation of incomplete bowel movement	4.0 (3.0, 5.0)	5.0 (3.0, 5.0)	0.0 (0.0, 1.0)	0.713

* Wilcoxon's signed‐rank test.

### Safety

Table 4 summarizes the adverse drug reactions that occurred during the study. Nausea was the most frequently reported adverse drug reaction (15.2%), followed by diarrhea (11.1%), gastrointestinal disorder, other (no improvement in bowel movements), and abdominal pain (5.1% each). The incidence of individual adverse events was the same as that of individual adverse drug reactions.

**Table 4:** Adverse drug reactions (safety analysis set) (*n* = 99)

Adverse drug reaction	Number of events	Number of patients (%)
Nausea	15	15 (15.2)
Decreased level of consciousness	1	1 (1.0)
Gastrointestinal disorder, other (no improvement in bowel movements)	6	5 (5.1)
Gastrointestinal disorder, other (no improvement in constipation symptoms)	2	2 (2.0)
Stomach pain	1	1 (1.0)
General and systemic disorders and conditions at the site of administration, other physical conditions	1	1 (1.0)
Diarrhea	11	11 (11.1)
Localized edema	1	1 (1.0)
Hemorrhoids	1	1 (1.0)
Headache	2	2 (2.0)
Abdominal pain	5	5 (5.1)
Abdominal distension	3	3 (3.0)
Constipation	3	3 (3.0)
Vomiting	4	4 (4.0)

## Discussion

This is the first study to investigate the influence of switching therapy to lubiprostone from a stimulant laxative among elderly patients with CC. Regarding the primary endpoint, there was no significant difference in the average spontaneous defecations per week from baseline (8.3) to Week 1 (7.8). However, significant differences were noted at Weeks 2 and 4, when the number of spontaneous defecations per week slightly decreased (mean change from baseline, −1.5 for both weeks). Although the number of spontaneous defecations significantly decreased, the frequency was within the clinically acceptable range throughout the study, as constipation is defined as fewer than three spontaneous defecations per week.^20^ These findings suggested that substituting lubiprostone monotherapy for a stimulant laxative at least resulted in the maintenance of a normal range of spontaneous defecations per week among elderly patients with CC, even after 4 weeks. It should be noted that at baseline in this study, the patients already had a mean of 8.3 spontaneous defecations per week. The median time to first spontaneous defecation after switching was 9.5 h, which is shorter than the 13.1 h reported for the Phase III clinical trial in Japan.^21^ The fact that the patients were on stimulant laxatives prior to switching likely explains the shorter time observed in the present study.

Neither the mean BSFS score nor the distribution of the BSFS changed significantly throughout the 4 weeks following the switch to lubiprostone. Most patients were within the normal score range of 3–5, both at baseline and Week 4. Thus, the potential impact of switching to lubiprostone on stool conditions was limited. A previous study in Japanese patients reported that around half of patients with CC undergoing treatment had hard stools with a BSFS score of 1 or 2.^22^ This indicates that the patients in the present study tended to have soft stools. Although there was no significant change from baseline to Week 4, there was a tendency toward hard stools at Week 2. No significant change was observed in the proportion of patients in the present study with a score of 4, which is considered ideal in Japanese patients.^23^

The PAC‐QOL questionnaire score increased during the study, indicating a decrease in quality of life. It is possible that scores increased after switching to a different treatment because most of the patients were satisfied with treatment with a stimulant laxative. This is supported by the fact that the patients had the normal frequency of defecation with stimulant laxatives. In addition, patients who took laxatives earlier experienced a decrease in drug intake after enrollment in the study, which may have affected their satisfaction. The decrease in PAC‐QOL, which includes treatment satisfaction, may have also been related to habituation caused by the prolonged use of stimulant laxatives.^12^ Switching to a drug with a different mechanism of action may affect other aspects of CC‐associated physiological conditions, such as depression or anxiety.^24^, ^25^ However, switching from stimulant laxatives to lubiprostone monotherapy did not appear to worsen psychological conditions in the current study, as patients in our study had no change in their psychosocial discomfort scores. Conversely, the scores of physical discomfort and worries/concerns decreased. This may indicate that changing from a stimulant laxative, which works by physically increasing colonic peristalsis, to lubiprostone monotherapy, which promotes water secretion in the small intestine, directly affects changes in defecation habits. When developing a switching treatment strategy from stimulant laxatives, it is necessary to confirm not only the frequency of defecation but also patient QOL.

The significant change in subjective symptom scores from baseline was observed only for nausea, which was the most frequently reported adverse event and adverse drug reaction, with a frequency of 15.2% each. In the Phase III study of lubiprostone, nausea was reported in 14.5% of patients, and 23.2% of the 315 safety evaluable patients at the time of approval (data from clinical studies conducted in Japan),^18^ suggesting that the incidence of adverse events and adverse drug reactions in the current study was comparable to that of previous studies.^26^ The results were also somewhat consistent with a retrospective study analyzing adverse event risk factors associated with lubiprostone, in which the salient adverse events were diarrhea (6.1%) and nausea (4.2%).^27^ In this study, three patients discontinued because of adverse events, and 14 patients underwent lubiprostone dose reductions. A recent study found that lubiprostone dose reduction to 12 μg twice daily minimized treatment discontinuation in patients with CC and did not affect efficacy.^28^ Treatment strategies with attention to side effects, such as nausea, are required to mitigate the discontinuation of lubiprostone treatment.

As noted, switching from stimulant laxatives to lubiprostone monotherapy is effective and poses no significant safety concerns. However, switching therapy should be undertaken with caution because a change in QOL or treatment satisfaction may be unacceptable for patients satisfied with the long‐term use of a stimulant laxative. In clinical practice, it may be more useful to have a treatment strategy such as a gradual dose reduction of the stimulant laxative while receiving lubiprostone and eventually transitioning to monotherapy than to switch to monotherapy at once.

The main study limitation was the single‐arm study design without a comparison group. Change based on the time before switching to lubiprostone monotherapy was evaluated, which requires a between‐group comparison for validation. The treatment status of each patient differed, as we did not specify the prescription status of stimulant laxatives (number of drugs and dosage) prior to enrollment in this study. Therefore, it is not possible to determine the influence of pre‐treatment status. The sample size (*n* = 99) was much smaller than the planned sample size of 150 patients, which limited the study in that a more detailed subanalysis could not be performed. The study duration was limited to 28 days; therefore, long‐term safety and effectiveness could not be evaluated. Finally, the study was conducted only in Japanese patients, potentially limiting the generalizability of the findings.

## Conclusions

Lubiprostone monotherapy was substantially effective and safe when used as switching therapy for elderly constipated patients treated with stimulant laxatives over 2 weeks. Dependence on long‐term stimulant laxatives might affect patient QOL and treatment satisfaction after switching therapy. Therefore, it is suggested that careful treatment strategies facilitating gradual switching to lubiprostone monotherapy may be needed in patients prescribed and satisfied with stimulant laxatives under real‐world clinical practice.

## Ethics approval

The third‐party Certified Review Board (CRB) of the Hattori Clinic reviewed and approved the protocol with the code CRB3180027. The CRB of the Hattori Clinic is a research review committee that is approved by the Japanese Ministry of Health, Labour and Welfare, in accordance with the Clinical Research Act. The role of the CRB is to review and approve clinical research conducted under this act. Participants gave informed consent to participate in the study before taking part.

## Data Availability

Due to privacy and ethical concerns, neither the data nor the source of the data can be made available.
